# Interaction of Spike protein and lipid membrane of SARS-CoV-2 with Ursodeoxycholic acid, an in-silico analysis

**DOI:** 10.1038/s41598-021-01705-5

**Published:** 2021-11-15

**Authors:** Francisco Javier Rodal Canales, Laura Pérez-Campos Mayoral, María Teresa Hernández-Huerta, Luis Manuel Sánchez Navarro, Carlos Alberto Matias-Cervantes, Margarito Martínez Cruz, Eli Cruz Parada, Edgar Zenteno, Edgar Gustavo Ramos-Martínez, Eduardo Pérez-Campos Mayoral, Carlos Romero Díaz, Eduardo Pérez-Campos

**Affiliations:** 1 Research Centre Faculty of Medicine UNAM-UABJO, Faculty of Medicine and Surgery, Autonomous University “Benito Juárez” of Oaxaca, 68020 Oaxaca, Mexico; 2 CONACyT, Faculty of Medicine and Surgery, Autonomous University “Benito Juárez” of Oaxaca, 68020 Oaxaca, Mexico; 3 National Technology of Mexico/IT Oaxaca, 68030 Oaxaca, Mexico; 4 Faculty of Medicine, National Autonomous University of Mexico, 04360 Mexico City, Mexico; 5 School of Sciences/Autonomous University “Benito Juárez” of Oaxaca, 68125 Oaxaca, Mexico; 6 Clinical Pathology Laboratory, “Eduardo Pérez Ortega”, 68000 Oaxaca, Mexico

**Keywords:** Computational biology and bioinformatics, Drug development

## Abstract

Numerous repositioned drugs have been sought to decrease the severity of SARS-CoV-2 infection. It is known that among its physicochemical properties, Ursodeoxycholic Acid (UDCA) has a reduction in surface tension and cholesterol solubilization, it has also been used to treat cholesterol gallstones and viral hepatitis. In this study, molecular docking was performed with the SARS-CoV-2 Spike protein and UDCA. In order to confirm this interaction, we used Molecular Dynamics (MD) in “SARS-CoV-2 Spike protein-UDCA”. Using another system, we also simulated MD with six UDCA residues around the Spike protein at random, naming this “SARS-CoV-2 Spike protein-6UDCA”. Finally, we evaluated the possible interaction between UDCA and different types of membranes, considering the possible membrane conformation of SARS-CoV-2, this was named “SARS-CoV-2 membrane-UDCA”. In the “SARS-CoV-2 Spike protein-UDCA”, we found that UDCA exhibits affinity towards the central region of the Spike protein structure of − 386.35 kcal/mol, in a region with 3 alpha helices, which comprises residues from K986 to C1032 of each monomer. MD confirmed that UDCA remains attached and occasionally forms hydrogen bonds with residues R995 and T998. In the presence of UDCA, we observed that the distances between residues atoms OG1 and CG2 of T998 in the monomers A, B, and C in the prefusion state do not change and remain at 5.93 ± 0.62 and 7.78 ± 0.51 Å, respectively, compared to the post-fusion state. Next, in “SARS-CoV-2 Spike protein-6UDCA”, the three UDCA showed affinity towards different regions of the Spike protein, but only one of them remained bound to the region between the region's heptad repeat 1 and heptad repeat 2 (HR1 and HR2) for 375 ps of the trajectory. The RMSD of monomer C was the smallest of the three monomers with a value of 2.89 ± 0.32, likewise, the smallest RMSF was also of the monomer C (2.25 ± 056). In addition, in the simulation of “SARS-CoV-2 membrane-UDCA”, UDCA had a higher affinity toward the virion-like membrane; where three of the four residues remained attached once they were close (5 Å, to the centre of mass) to the membrane by 30 ns. However, only one of them remained attached to the plasma-like membrane and this was in a cluster of cholesterol molecules. We have shown that UDCA interacts in two distinct regions of Spike protein sequences. In addition, UDCA tends to stay bound to the membrane, which could potentially reduce the internalization of SARS-CoV-2 in the host cell.

## Introduction

SARS-CoV-2 is a single-stranded RNA beta coronavirus, which causes COVID-19. The urgency of developing effective drugs for the COVID-19 treatment has been of the utmost importance^1^. Great interest has been generated to identify targets for drug repurposing by maps of proteins interactions with SAR-CoV-2^2^, and many come from natural products of plant origin^3–6^. According to the information released, as of November 5, 2021, SARS-CoV-2 has caused more than 248 million infections worldwide and more than 5 million deaths^7,8^. COVID-19 manifests itself through heterogeneous clinical expression, with respiratory, cardiovascular, nervous system, liver and skin symptoms^9^.

SARS-CoV-2 is surrounded on average by 40 to 48 Spike (S) trimer proteins^10,11^, these are heavily glycosylated and have three receptor-binding domains (RBDs), covered on top of the S trimer by the N-terminal domains (NTD)^12,13^. These are mainly found in a closed prefusion conformation^8^. RBDs are exposed or move up the viral membrane in their closed conformation. The Spike protein, being connected to the membrane through a stalk region that has three flexible hinges, allows protein S to be dynamic^5^. Between the trimeric region and the virion membrane, there is a triple-stranded coiled-coil heptad repeat 2 (HR2) with three flexible hinges in the stem region, which can allow lateral displacement of the stalk^8,12^. The S trimer of SARS-CoV-2 binds to its receptor ACE2 for subsequent membrane fusion and virus entry. Cleavage of protein S by furin and/or TMPRSS2 proteases is key to this binding^14^. The “fusion proteins” have an α-helical, coiled-coil structure, which requires proteolytic processing to generate a structure of fusion^15^.

Recently, it has been reported that SARS-CoV-2 budding is found in regions close to the endoplasmic reticulum and Golgi-like membrane arrangements^10^, due to the composition of the endoplasmic reticulum Golgi intermediate compartment cisternae (ERGIC), it has been proposed as the main composition of the virus membranes^16^. ERGIC is composed of approximately 58% phosphatidylcholine, 22% phosphatidylethanolamine, 3% phosphatidylserine, 10% phosphatidylinositol, 3% sphingomyelin, and 0.08% cholesterol^17^.

In the case of COVID-19, Ursodeoxycholic acid (UDCA), also called ursodiol, is a repurposed drug that has been suggested for clinical trials due to its properties in modulating apoptosis and the hyper-immune inflammatory response^18,19^.

UDCA is a bile salt, it is amphipathic, thus, it has a hydrophobic component that is soluble in lipids and a hydrophilic component that is soluble in water. UDCA is also known as 3α, 7β-dihydroxyβ-cholan-24-oic acid. It is not synthesized by the liver, it originates in the colon from bacterial epimerization 7β (from *Ruminococcus gnavus* N53 in human gut microbiota) from primary bile to be absorbed later in the colon^20,21^, this represents 4% of bile acids^22^.

Although UDCA is recognized as a non-antiviral hepatoprotective agent, it is used in combination with antivirals for the treatment of some viral diseases, such as viral hepatitis^7^. Also, UDCA alone or in combination with antivirals or immunomodulators, improve patients with hepatitis A virus (HAV), hepatitis B (HBV), hepatitis C (HCV), hepatitis D (HDV), hepatitis E (HEV), and Non-A-E acute viral hepatitis^23,24^. In addition, potential beneficial effects in acquired immunodeficiency virus have been shown in pilot studies with UDCA^25^. Previously, Yadav et al.^26^ have explored the effect of ursodeoxycholate and chenodeoxycholate against SARS-CoV-2 envelope protein. They found that these molecules increase permeability just like surfactants/detergents.

The repositioning of old drugs arises as an advantage due to their previous development and application over the years^27^. Among the methods used in drug repurposing studies are molecular docking and dynamics simulations^28^. Molecular Dynamics is one of the computational strategies used for predicting the complementarity of the binding site between the ligand and the target^29,30^.

In order to show the interaction of the SARS-CoV-2 virus with UDCA, we took into account the UDCA characteristics of hydrophobicity and its action on cholesterol. Here, we explored the interaction between both UDCA and the Spike protein through in silico studies, as well as UDCA in the presence of a model of the membrane of SARS-CoV-2. Also, according to the characteristics of the lipid constituents, we constructed different types of membrane and recorded possible UDCA/membrane interactions.

## Materials and methods

### Design and data set

For the "SARS-CoV-2 Spike protein-UDCA" analysis, docking and MD were applied to confirm the interaction between the SARS-CoV-2 Spike protein and UDCA. For the "SARS-CoV-2 Spike protein-6UDCA", we used six UDCA residues around the Spike protein and performed MD. In addition, for the "SARS-CoV-2 membrane-UDCA", we evaluated the interaction of UDCA with different types of membranes, including one with similar characteristics to the SARS-CoV-2 membrane.

The structure of UDCA was obtained from the Protein Data Bank (PDB)^31^, and its force fields were obtained with “reduce” and “antechamber”, utilities found within the AMBER14 package^32^. The trimeric structure of the Spike protein was taken from code 6VSB.pdb^33^. The membrane model was designed and built as indicated below. Notably, we carried out MD with 7DF3.pdb^34^ in a prefusion state, and we obtained the same results. To visualize MD´s trajectories, we used UCSF CHIMERA V.1.15^35^, and VMD 1.9.3^36^.

### Docking study

We analysed the interaction between UDCA^37^ and the trimeric structure of the Spike protein using HEX 6.3 software [Hex Protein Docking, loria.fr]^38^ through geometric correlation, with an energy minimization post-processing. The dimension of the grid was 0.75 and steric scan 18, a random seed, receptor and ligand range of 180°, and a range of rotation of 360°.

### Molecular dynamics simulation with SARS-CoV-2 Spike protein and UDCA

The "SARS-CoV-2 Spike protein-UDCA" was obtained by docking a structure with lower energy, which corresponded to − 386.35 kcal/mol. Next, the complex was subjected to MD with AMBER14 software^27^. We did a minimization of 25,000 steps, with bond length restrictions for hydrogen atoms (ntc = 2), a cut-off radius of 8.0 Å, and positional restrictions (restraint_wt = 2). The structure was heated from 0 to 300 K for 250,000 steps with a 0.002 ps time step and bond length and positional constraints, a nonbonded cut-off of 8.0 Å, and using a Langevin thermostat. Then, density balancing was done over 750,000 steps with a time step of 0.002, 8.0 Å nonbonded cut-off, 1.0 pressure relaxation time, Langevin thermostat for temperature control, a collision frequency of 2.0/ps (gammaln = 2.0) and positional constraints. Finally, a molecular dynamic of 4,000,000 steps (total 10 ns) was executed with a time step of 0.002 ps, a cut-off radius of 8.0 Å, Langevin thermostat for temperature control (ntt = 3) at 300 K, a collision frequency of 2.0 (gamma_ln = 2.0) and positional constraints.

For the "SARS-CoV-2 Spike protein-6UDCA", we surround the trimeric structure of the Spike protein with six UDCA residues through MD. The same parameters were used for minimization, state solvation, heating, density balance and production of molecular dynamics. All sugar residues attached to Spike protein were removed. The trajectory analysis was visualized with VMD 1.9.3^37^ and the distance and hydrogen bond calculations were carried out with CPPTRAJ^39^.

### Membrane design

**Figure 1:**
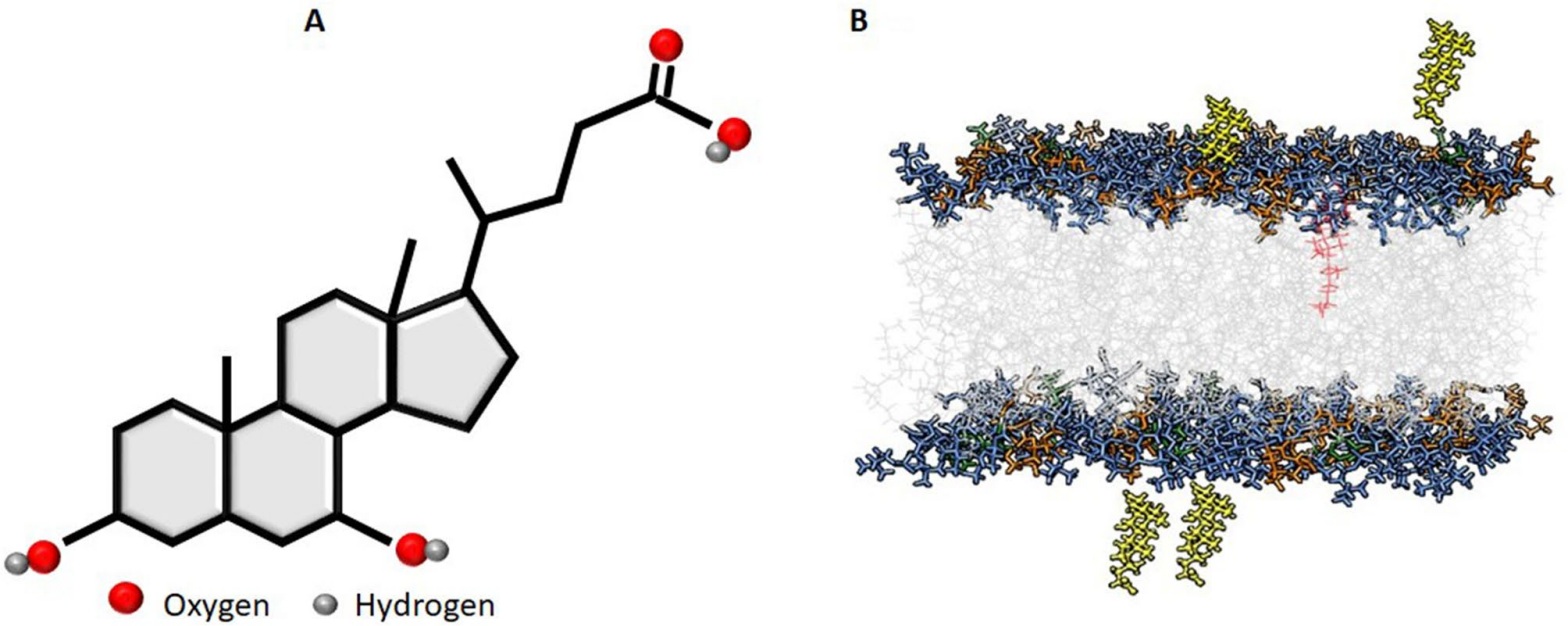
UDCA structure and identification and distribution of UDCA in the different membranes. (**A**) UDCA schematic representation, using the SMILE code (CC(CCC(= O)O)C1CCC2C1(CCC3C2C(CC4C3(CCC(C4)O)C)O)C). (**B**) Membrane ERGIC-like. All membranes (VIR, ERGIC, and PLASM) had a similar UDCA spatial distribution. This figure shows an ERGIC-like membrane built with Charmm-gui [CHARMM-GUI] server. Image edited with UCSF Chimera V 1.15. Grey lines: glycerol, orange lines: PE, blue lines: PC, green lines: PS, red lines: CHL and yellow lines: surrounding UDCA residues.

Considering that SARS-CoV-2 buds in regions with a high vesicle density besides the endoplasmic reticulum (ER) and Golgi-like membrane^10^, we designed different membranes, including a SARS-CoV-2-like membrane (ERGIC-like). Virus (VIR)^40^, ERGIC with cholesterol^14^, and eukaryotic plasmatic membranes (PLASM) with cholesterol^24^ were constructed (Table 1). The same phospholipid distribution was used in all membranes and four UDCA residues were added, two in each leaf (Fig. 1). Thus, in "SARS-CoV-2 membrane-UDCA", we observed the response of four UDCA residues around the ERGIC-type, virion-type and plasma-type bilipid membranes. We constructed a model of six lipid bilayer membranes, three for reference, using dioleoyl-phosphatidic acid (DOPA), dioleoyl-phosphatidylcholine (DOPC), dioleoyl-phosphatidylethanolamine (DOPE), dioleoyl-phosphatidylserine (DOPS), and cholesterol (CHL) (128 each one, 64 in each leaf).

**Table 1:** The number of phospholipids in each membrane.

Phospholipid	Virus	ERGIC	PLASM
DOPA	4	0	0
DOPC	7	40	21
DOPE	35	18	11
DOPS	18	5	8
CHL	0	1	24
TOTAL	64	64	64

*DOPA* dioleoyl-phosphatidic acid, *DOPC* dioleoyl- phosphatidyl choline, *DOPE* dioleoyl-phosphatidyl ethanolamine, *DOPS* dioleoyl-phosphatidyl choline, *CHL* Cholesterol, *ERGIC* endoplasmic reticulum golgi intermediate compartment, *PLASM* plasmatic membrane.

We designed the following membranes: VIR, VIR-IU5, ERGIC, ERGIC-IU5, PLASM, PLASM-IU5, where IU5 denotes UDCA. Additionally, for comparison, we built two membrane systems with the same amount of phosphatidylcholine (PC), phosphatidylethanolamine (PE), phosphatidylserine (PS), and cholesterol, however, instead of using DOPC, DOPE and DOPS, we used POPC (1-palmitoyl-2-oleylphosphatidylcholine), POPE (1-palmitoyl-2-oleylphsphatidylethanolamine), and POPS (1-palmitoyl-2-oleylphosphatidylserine). The percentage of PC, PE, PS, and CHL is shown in Table 1. Sphingomyelin was not used in the systems because AMBER lacks force fields for these phospholipids.

The membrane systems were built with CHARMM-GUI^41,42^ using an average of 35 water molecules per lipid. To equalize the charges to zero, we employed symmetric lipidic bilayers with potassium ions with the force fields lipid17, TIP3, GAFF, and NTP assembly, in AMBER format.

Minimizations of 50,000 steps were performed at constant volume, no pressure scale, no restriction on hydrogen atoms and a nonbonded cut-off of 10.0 Å. After this, a preheating from 0 to 100 °K, 250,000 steps, with a 0.002-time step, hydrogen bond length restrictions, no evaluation for hydrogen bonds, relative geometric tolerance for coordinates of 0.0000001, Langevin thermostat and a collision rate of 1.0/ps. Then, we simulated a warm-up from 100 to 300 °K with the same parameters as in the previous preheat but for 250,000 steps, periodic boundary conditions at constant pressure, anisotropic pressure coupling, and a pressure relaxation time of 2.0 ps, all according to the AMBER14 instructions^43^. For the density balance, periodic limit conditions were used at constant pressure, Langevin's thermostat, a collision frequency of 1.0/ps, relative geometric tolerance for coordinates of 0.0000001, a cut-off radius of 10.0 Å during 1, 750,000 steps with a time step of 0.002 (for a total of 3.5 ps). The molecular dynamics was done during 60,000,000 steps with a time step of 0.002 (total 120 ps), with restrictions for hydrogen bonds, relative geometric tolerance for coordinates of 0.0000001, Langevin thermostat, with a collision frequency of 1.0/ps at temperature 300 °K, 10 Å cut-off, anisotropic pressure coupling, and periodic boundary conditions at constant pressure.

We used VMD 1.9.3^36^ for the visualization of the trajectories and CPPTRAJ^39^ for the calculation of distances, RMSD, and the calculation of hydrogen bonds.

## Results

### Interaction between the trimeric structure of the Spike protein and UDCA by docking

In the "SARS-CoV-2 Spike protein-UDCA" analysis, we employed the trimeric structure of the Spike protein and UDCA using the HEX 6.3 program. UDCA showed an affinity energy of − 386.35 kcal/mol with the residues conforming the three alpha central helices region. This set of three alpha helices is formed by the contribution of one alpha helix of every monomer (residues K986 to C1032). We think that this set of three alpha helices hold together the three monomers of the Spike protein before a post-fusion conformation.

**Figure 2:**
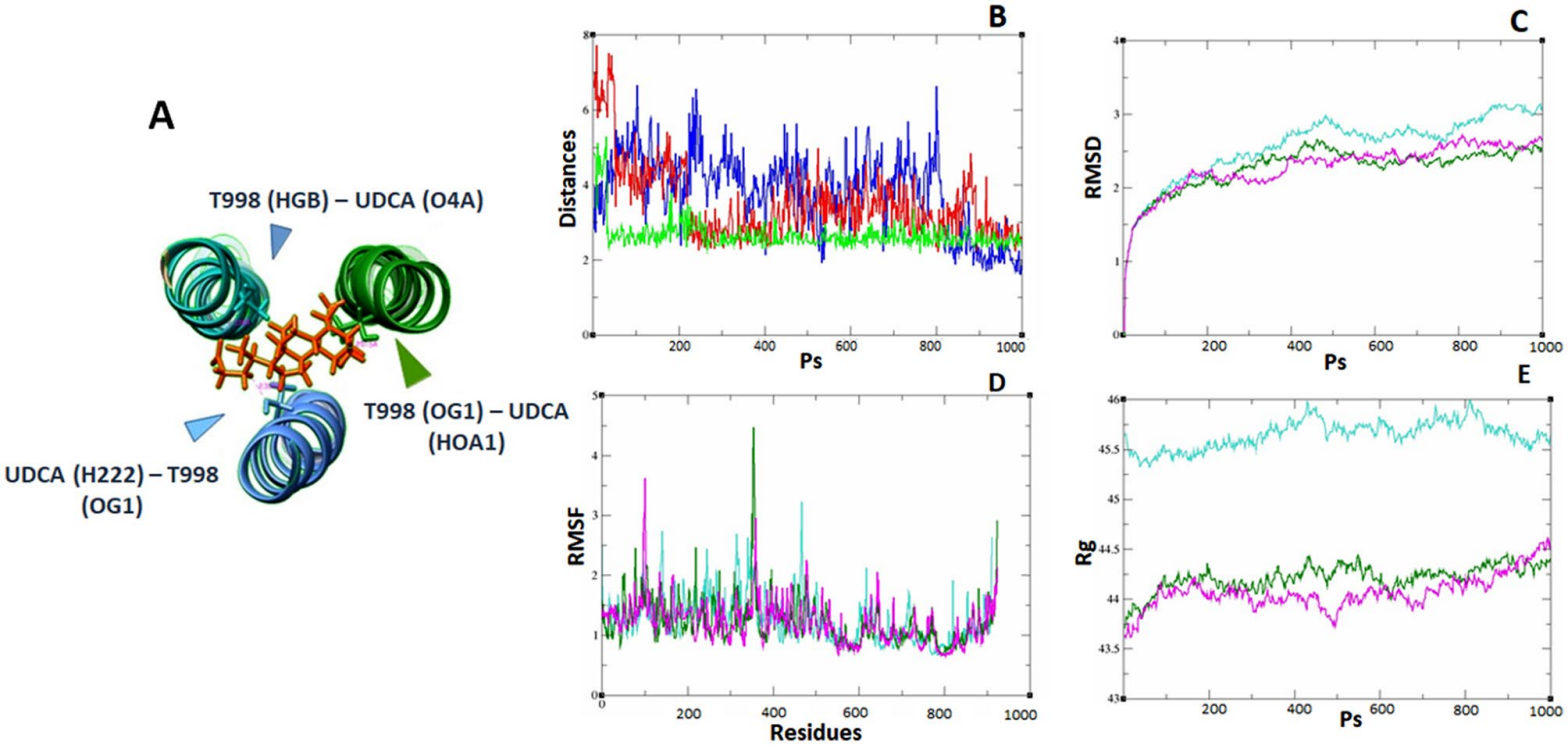
Schematic representation of UDCA in the central region of the alpha-helices. The UDCA—Spike protein interaction is maintained along the molecular dynamics’ trajectory due to the interaction of the bonds between the THR 998 atoms of monomers A, B, and C with UDCA. (**A**) UDCA interaction is shown with monomers A, B and C. UDCA in orange. Image edited with UCSF Chimera V 1.15. (**B**) The distances of the atoms that maintain this interaction are observed, the intervening atoms are shown in Table 2; blue line UDCA-T998 (chain B), red line UDCA-T998 chain C and green line UDCA-T998. (**C**) RMSD calculated by monomer (A, turquoise; B, green and C, magenta line) which shows stability in the simulation. (**D**) RMSF calculation, fluctuation of each residue per monomer (A, turquoise; B, green and C, magenta line) in the region of alpha helices, from lysine 986 to cysteine 1032. (**E**) Calculation of the radius of gyration that shows the degree of compactness of the monomers: A (45.64 ± 0.14; turquoise line) > B (44.21 ± 0.13; green line) > C (44.07 ± 0.17; magenta line).

When applying MD to this structure (spike protein-UDCA), we observed that the UDCA remained in the same position during the whole trajectory, and there were even moments when hydrogen bonds were formed with residues close to it (Table 2). UDCA had affinity mainly with residues R995 and T998 of monomers A, B, and C. Residues R995 and T998 were found in the central helices from subunit S2 (S2) of the protein (Fig. 2).

**Table 2:** Interactions between UDCA and T998 of central helices of S2 of Spike protein of SARS-CoV-2.

Frame	Acceptor	Donor		RMSF	Distance (nm)—angle (°)
41	T998@OG1 (A)	IU5_2906@HOA1	IU5_2906@O1A	0.77	2.8286 153.1908
3	IU5_2906@O4	T998@HG1 (C)	T998@OG1 (C)	0.79	2.8305 160.6405
3	IU5_2906@O4A	G999@H (B)	G999@N (B)	0.79	2.9078 140.9356
2	IU5_2906@O1B	R995@HH12 (C)	R995@NH1 (C)	0.94	2.8931 151.5614
1	D994@OD2 (A)	IU5_2906@HOA1	IU5_2906@O1A	0.74	2.5852 154.5614
	D994@OD2 (A)	IU5_2906@HOB1	IU5_2906@O1B	0.74	2.8389 162.9852
	D994@O(A)	IU5_2906@HOA1	IU5_2906@O1A	0.74	2.8648 147.6706
	IU5_2906@O1A	R995@HH12 (A)	R995@NH1	0.94	2.8746 136.8333
	R995@NH1 (C)	IU5_2906@HOA1	IU5_2906@O1A	0.94	2.9528 167.5659

These distances and angles correspond to H bonds formed during the trajectory of molecular dynamics. As observed in Fig. 2B, once these atoms are at a close distance, they remain together along the trajectory. In molecular dynamics, most of the H-bonds are at the beginning of the trajectory. H-bonds are formed between UDCA (denoted as IU5_2906 according to AMBER files) and T998, R995, D994 and G999 from three monomers (H-bonds obtained with CPPTRAJ).

**Figure 3:**
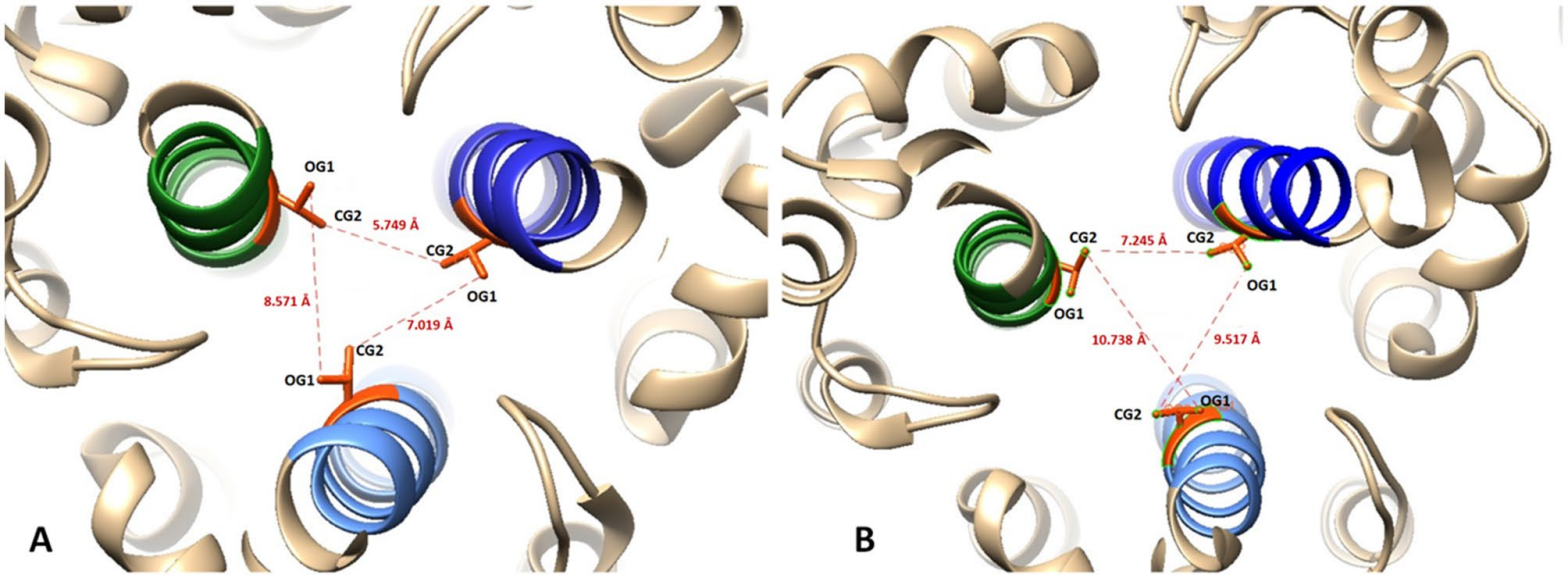
Distances between OG1 and CG2 of the monomers A (green), B (blue), and C (light blue) of THR 988 in Spike protein. We observed that there is a slight increase in distance suggesting that these helices could separate from each other once the membrane fusion process begins. In (**A**) the closed conformation is observed (smaller distances) and in (**B**) the open conformation. With the presence of UDCA, it remains in a conformation similar to (**A**). Taken from 7DF3.pdb and 7BNO.pdb and edited with chimera V. 1.15.

The oxygen atom OG1 of T998 of the monomer A interacts with the hydrogen HOA1 of UDCA. The hydrogen atom HGB of T998 of monomer B interacts with the oxygen atom O4 of the UDCA, and the oxygen atom OG1 of the residue T998 of monomer C interacts with the hydrogen H222 of UDCA with average distances of 2.67 ± 0.41 Å. On the other hand, the distances between monomers A, B, and C in the pre-fusion state were 5.6 and 8.6 Å for OG1 and CG2 of every T998, respectively, and in the post-fusion state, it is 9.58 and 9.23 Å for the same atoms (Fig. 3A,B). In presence of UDCA, these distances are not modified and remained at 5.93 ± 0.62 and 7.78 ± 0.51 Å, respectively (Fig. 2A). This leads us to consider that UDCA could act as a connecting bridge that would not allow an adequate conformational shape for the post-fusion state. The RMSD, RMSF as well as Rg of monomers A, B, and C were maintained stable as shown in Fig. 2B–E, indicating an equilibrated system. The monomer C showed the smallest RMSD compared with monomer A and B (RMSD (A), 4.03 ± 0.6 Å; RMSD (B), 3.14 ± 0.42 Å and RMSD (C), 2.89 ± 0.32 Å). RMSF average of monomer C was 2.25 ± 0.56 and the average Rg of monomer C was 44.31 ± 0.28.

Likewise, the distances between UDCA and residues R995, T998 and D994, R995 and T998 of the second and third monomers were preserved. In Table 2, we observed that ARG 995, THR 998 and GLY 999 atoms showed a low RMSF (0.94, 0.77 and 0.79, respectively). The lowest RMSF of THR could explain the interactions established with UDCA since it had a lower value and major stability.

Furthermore, we compared the closed (pre-fusion) and open (post-fusion) conformation of the alpha-helices of the Spike protein^34,43^ using UCSF CHIMERA V.1.15^36^. We observed that in the closed conformation (7DF3.pdb) (Fig. 3A), the OG1 and CG2 atoms of every T998 are separated for 5.6 and 8.6 Å from each other, respectively. However, for the open conformation (7BNO.pdb) (Fig. 3B), those same atoms had a distance of 9.58 and 9.23 Å, respectively. This suggests that in post-fusion conformation, these alfa-helices are slightly separated, and this separation is important for correcting fusion between viral and host membranes; although, in the presence of UDCA, we observed that the conformation remains closed, as seen in Fig. 2A. In accordance with the observed distances, we think that if UDCA binds to this region and serves as a bridge between these residues, it could prevent the Spike protein from folding correctly to its open conformation, and thus not carry out membrane fusion, Fig. 2A.

### UDCA showed affinity towards a region between HR1 and HR2

**Figure 4:**
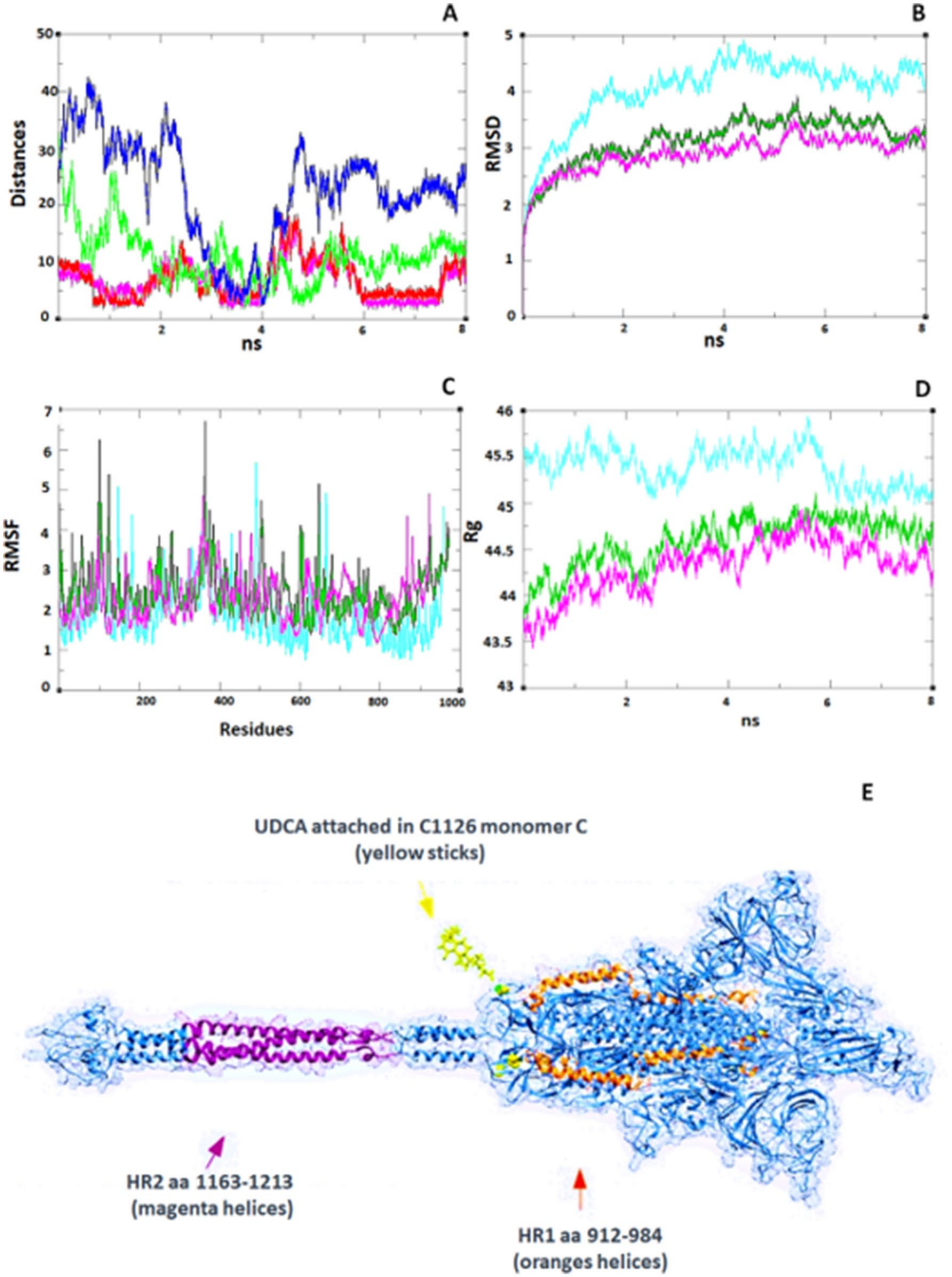
Interaction of UDCA with cysteine residue C1126. (**A**) Distances between UDCA 2908 and C1126 of monomer C (atom O4-UDCA vs HB2-C1126. Atom O4A-UDCA vs HB3-C1126 in magenta and red, respectively). UDCA-2916 interaction with N536 (atom HC11-UDCA vs OD1-N536 in green line). UDCA 2912 interaction with T602 (O4-UDCA vs HB-T602 in blue line). Residues N536 and T602 bind to UDCA momently, instead C1126 binds to UDCA and tends to retain it, thus we think that if UDCA interferes in this region near to HR1-HR2, it could prevent the coiled-coil heptad repeat binding in the area between the HR1 and HR2 regions, necessary for membrane fusion. (**B**–**D**) (monomer A in turquoise line, B in green line and C in magenta line), respectively. (**E**) General Overview of the Spike protein (taken from charm-gui.org, 6VSB.1.1.1). The HR1 regions of each of the A, B, and C monomers are shown in orange tones. The HR2s are shown in purple tones and the regions to which the UDCA binds in the simulations are shown in yellow (stick representation). Values of RMSD, RMSF and Rg indicates a balanced simulation, although there is a slightly higher value for monomer A, B, and C are very similar. The three UDCA in the MD were differentiated using the Amber nomenclature as UDCA-2908, UDCA-2916 and UDCA-2912.

In the "SARS-CoV-2 Spike protein-6UDCA", we placed six UDCAs around the trimeric structure of the Spike protein in its closed conformation (6VSB.pdb). We visually analyse the trajectory using VMD 1.9.3. We found that three of the six residues were attracted to three different regions of the Spike protein in different areas to the first analysis. One of them was attracted to an ASN536 residue of the C monomer for a very short period of 50 ps, while the rest of the trajectory was kept repelled. Another UDCA was attracted to a residue of THR of chain C with a very short period of 25 ps. The third one was attracted by CYS1126 of chain C with a longer period. The last interaction UDCA-CYS1126 is conserved in different periods of the trajectory. Also, these residues interact for long periods of time, for up to 375 ps, which suggests a strong attraction. The distances between UDCA and N536 was 10.94 ± 4.69 Å, between UDCA and T602 was 10.94 ± 4.69 Å, and between UDCA and C1126 was 6.99 ± 3.15 Å. In monomer C, we found 1.39 of RMSF with CYS1126, less than the average (1.81 ± 0.55) of the whole monomer C; monomer A with 4.03 ± 0.6, monomer B with 3.14 ± 0.42 and monomer C with 2.89 ± 0.32. The RMFS's and Rg of each monomer were similar (Fig. 4A–D). A graphical representation is shown in Fig. 4E, where we see the UDCA interacting with the region between HR1 and HR2.

### Lipid membranes and UDCA

**Figure 5:**
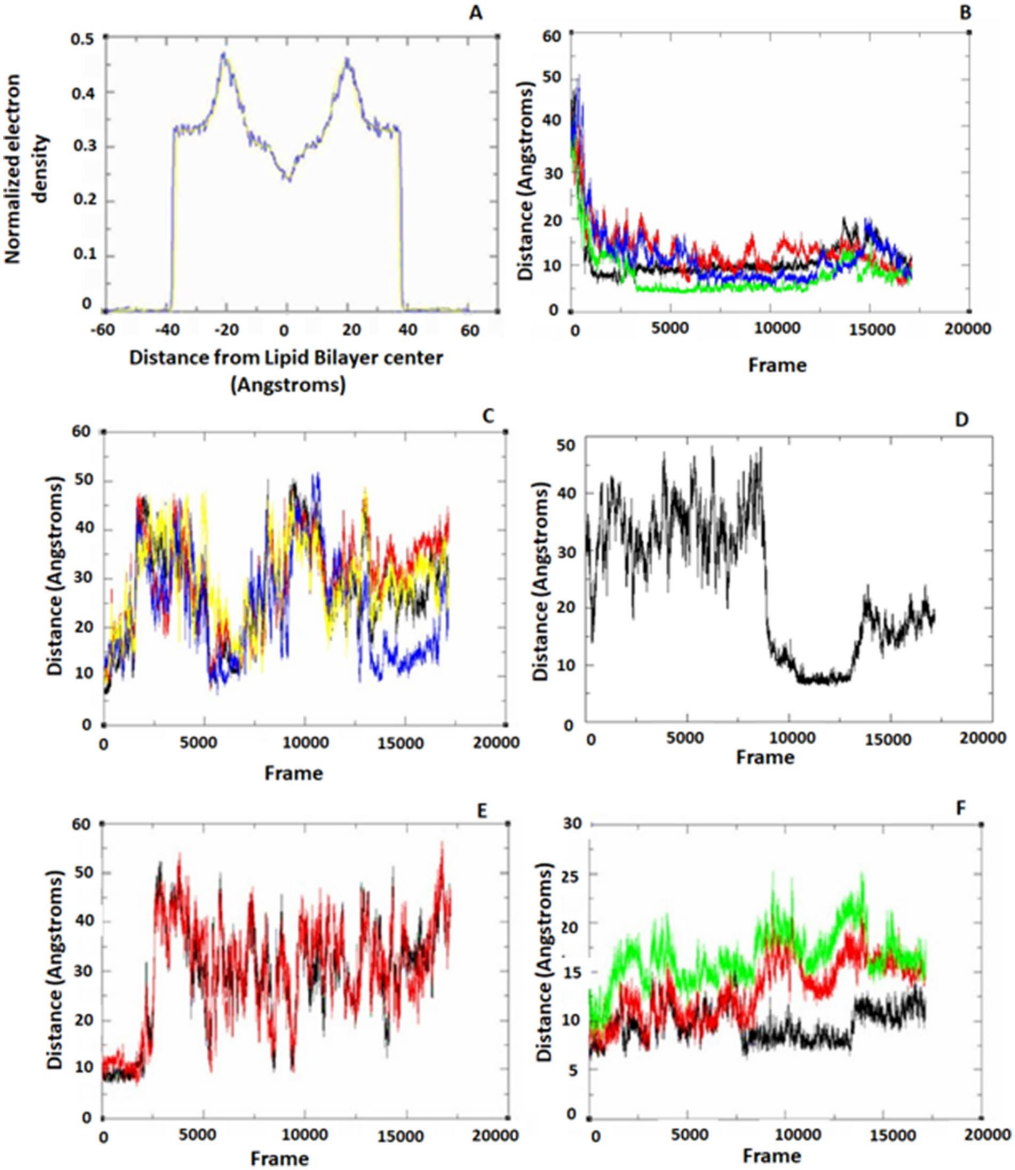
Electron density profile bilayer membranes and distances between the centre of mass of UDCA and PE. (**A**) Electron density profile bilayer membranes was very similar in all membranes along the trajectory, showing stable Molecular Dynamics. The virion membrane has a major percentage of DOPE and DOPS. The electron density remains stable on both leaves of the membrane (± 20 Angstroms). (**B**) Distances from the centre of mass of UDCA in relation to four centres of mass of PE, PE272 (black), PE218 (red), PE251 (green) and PC221 (blue), to which the UDCA molecules were attracted. We observed that in the virus membrane, once the UDCA residue is attracted, it tends to remain attached to the membrane. Distances calculated with CPPTRAJ. Also, in the Golgi intermediate compartment membrane (ERGIC), we have shown the distances from the centre of mass of UDCA residues to the phospholipid residues, for which it exhibits affinity. (**C**) It is observed that residue 401 of UDCA is close to the phospholipids PE17(black), PE20 (red), PC59 (blue) and PC47 (yellow). (**D**) The distance of residue UDCA 403 towards PE293 (black line) is observed. Both figures show affinity, however, they do not remain together over time**.** In addition, we evaluate the distances between the UDCA residues and PC, PE and CHL phospholipids. (**E**) We observed the distances between UDCA and PE. It is noteworthy that the only way that UDCA was attached to cholesterol was because 3 cholesterol residues were found together, if they were separate, UDCA and cholesterol tended to separate. (**F**) The distances between residues of UDCA and cholesterol are shown, we observed that from the beginning the UDCA and PE/CHL were united but over time they tended to separate.

**Figure 6:**
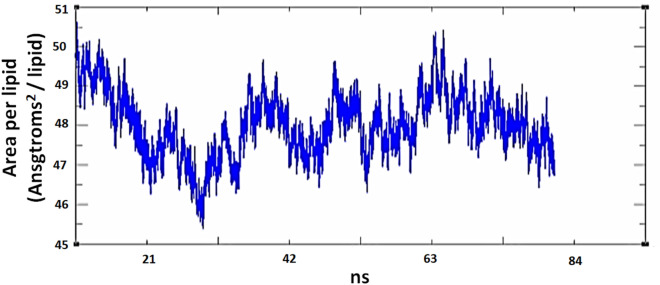
Area per lipid for PLASM-IU5-like membrane. As shown in the graph, it tends to be stable along the trajectory. This parameter was similar for all of the rest of the membranes simulated. Obtained with CCPTRAJ.

In "SARS-CoV-2 membrane-UDCA", we used dioleoyl- or palmitoyl- as controls and observed that the electron density remained stable on both sides of the membrane, in a range of ± 20 Å along all trajectories (Figs. 5A and 6), indicating balanced simulations.

Three UDCAs were attracted to the virion-like membrane, which was retained for a long time (30 ns), as was observed in the distances of the centres of mass of the UDCAs with respect to the PC and PE. UDCA bound to these residues from the beginning of the trajectory and remained together along the trajectory (Fig. 5B). In the Supplementary files, we show the simulation of a membrane virion-like with UDCA.

In the ERGIC-like membrane, only one leaf contained a cholesterol residue, the other side contained 62.5% PC, 28.2% PE and 8% PS. Possibly because of cholesterol, the UDCAs that were attracted to the membrane only remained attached for a short time and then were detached, as shown in Fig. 5C,D.

In a eukaryotic PLASM-like membrane, there was 37,5% CHL, 33% PC, 17% PE, and 12,5% PS. We found that two UDCA residues maintained a close distance to the phospholipids, Fig. 5E,F. However, they did not stay together along the membrane. The behaviour of PC and PE is the same, they tend to attract UDCA residues. It is notorious that in the PLASM-like membrane, a cluster of three cholesterols is formed (charmm-gui.org server aleatory constructed that way), and only this way, cholesterol attracted UDCA, since if cholesterol is found alone, it does not attract UDCA.

It is noteworthy that UDCA does not show attraction to membranes containing palmitoyl (phospholipids with 20 carbons), whatever its constitution.

## Discussion

### UDCA interacts in the central helix region of S2 and HR1 and HR2

We observed that UDCA interacts in two regions of Spike protein. One of the regions which shown attraction is in the area with three alpha-helices of three monomers of the Spike protein (residues R995, T998, V991, D994, T998, T998, and R995), and the other, with a CYS1126 residue in a zone between the HR1 and HR2 regions.

Also, we compared the pre-fusion and post-fusion conformation of the alpha-helices of the Spike protein^34,36,43^ and observed that in the pre-fusion conformation (7DF3.pdb, Fig. 3A) and the post-fusion conformation (7BNO.pdb, Fig. 3B), those same atoms have a distance of 9.58 and 9.23 Å, respectively. As was measured before^44^ in post-fusion conformation, these alfa-helices are slightly separated; this separation is important for a correct fusion between viral and host membranes, but in the presence of UDCA, we observed that the conformation remains closed.

This leads us to consider that UDCA could join in two regions of the Spike protein, in the S2 region, at residue T998 found in the central helices formed by residues K986-C1032. Precisely in this region, there are two mutations of interest, the K986 and V987, these are already being induced with proline substitutions in several vaccines that are in development^45^. In the joint of the central helices of S2, UDCA could prevent conformational changes in the Spike protein once it binds to ACE2. UDCA could act as a link between the three monomers, since as we observed in Fig. 2A,B, UDCA forms hydrogen bonds with the T998 residues of each of the monomers.

### UDCA interacts in the central helix region of S2 and HR1 and HR2

A key step in the coronavirus infection process is membrane fusion^46^. In this stage, the virus protein fusion core is formed by the union of three copies of HR1 and three of HR2. This stage had been verified in a certain way by the inhibition of fusion through the introduction of peptides, analogous to HR1 and HR2. According to the current model, HR1 and HR2 are joined to form the coiled-coil bundle necessary for fusion^46,47^.

The post-fusion stage takes place after the receptor-binding domain (RBD) has contact with its target protein (ACE2). After this contact, the Spike protein has a series of conformational changes, among these, the union of the three HR1 and three HR2^48^. Another region where the UDCA has a high probability of binding is an intermediate region located between the monomers HR1 and HR2, since it is more exposed, as seen in Fig. 4C. In this region, UDCA interacts with the residue C1126. This residue is part of the C1082-C1126 disulphide bridge, which could modulate the flexibility of the protein^49,50^.

The residue C1126 is in the region between this HR1 and HR2 and in our results of "SARS-CoV-2 protein-6UDCA", the oxygen atoms O4 and O4A of UDCA stays bound to the hydrogen atoms HB2 and HB3 of C1126, respectively, up to 375 ps. UDCA has a β-hydroxylation that provides it with greater hydrophilicity and thus could interact with hydrogens^51,52^. This causes that UDCA and cysteine C1126 to suddenly separate, however, nanoseconds later, both molecules tend to bound, as shown in Fig. 4A (red and magenta line).

### UDCAs interact in a stable time interval with the virion-like membrane and PLASM-like membrane

In membranes, UDCA interacts with PE and PC of virus-like and ERGIC-like membranes. Although with the virion-like membranes the interaction show more affinity, due to three of four residues are attracted to it, whereas in ERGIC-like membrane, only show that one UDCA is attracted.

The finding that three cholesterol molecules are present and UDCA is easily attracted, is related to published studies stating that eukaryotic membranes are rich in cholesterol, which is used by SARS-CoV-2 to enter cells, as it depends on a cholesterol-rich lipid raft^53^. Previously, another study found that cholesterol increases interactions with viral membranes due to its electrostatic and solvation properties^54^, moreover, cholesterol is also known to promote oligomerization of the SARS-CoV-2 FP^55^. Therefore, we think that if UDCA binds to cholesterol lipid rafts in eukaryotic cells, UDCA could hinder the interaction between cholesterol and FP and avoid oligomerization. According to Fig. 5A,B, UDCA may form a cluster with cholesterol-rich lipid rafts in eukaryotic membranes, and thus prevent that the fusion peptide of the Spike protein of SARS-CoV-2 from oligomerizes.

In Fig. 5A,B, we observed that in virion-like membranes, the distance of the centres of mass between UDCA and PE, at the beginning of the simulation are far apart, although they tend to attract each other and once they are joined, they no longer separate. Whereas ERGIC-like membrane, the distance from centres of mass of UDCA towards PE, shows that they are not attracted, except when three cholesterols are together, the UDCA tends to be attracted (PLASM-like membrane, Fig. 5A–F).

Likewise, bile acids are endogenous inhibitors of the NLRP3 inflammasome via NLRP3 ubiquitination, through the TGR5-cAMP-PKA axis. Bile acids could act through this pathway and regulate the activation of the NLRP3 inflammasome^56^ and attenuate COVID-19^57^. Furthermore, it has been reported that UDCA presents anti-apoptotic activity by modulating mitochondrial membrane permeability transition^58^.

However, within the limitations of this work, we were unable to build a more realistic membrane due to the lack of force fields of various lipids. In addition, the sugars that accompany the Spike protein could serve as protection against various compounds including UDCA. Therefore, removing them for simulation reduces reliability.

In conclusion, the interaction between UDCA and various residues of the Spike protein and its membrane indicates that UDCA has an affinity and remains attached to the central helix of S2 of the Spike protein monomers. UDCA interacts in a zone between HR1 and HR2 regions, it also tends to remain membrane-bound in the presence of cholesterol molecules. The data showed that UDCA could interact with the SARS-CoV-2 Spike protein and a membrane model like SARS-CoV-2, destabilizing the interaction of the virus its target cells.

## Supplementary Information

Supplementary Information.
