# Supplementary material for: Interaction of Spike protein and lipid membrane of SARS-CoV-2 with Ursodeoxycholic acid, an in-silico analysis
Source: Sci Rep. 2021 Nov 15;11:22288. doi: 10.1038/s41598-021-01705-5 (PMC8593036; doi:10.1038/s41598-021-01705-5)
Supplement: Supplementary file 1 — Supplementary Information. [file 41598_2021_1705_MOESM1_ESM.docx]

**Supplementary files.**

<https://u.pcloud.link/publink/show?code=XZHIQ1XZYETYJzwQR9LUrHteyzo6mjSUP3jV>

We show the simulation of a membrane whose composition is similar to the coat of a virion (54.6% DOPE, 11% DOPC, 21.1% DOPS, and 6.2% DOPA) by UDCA.

In the first two seconds, the lipid membrane is observed in an orthogonal box of water. Subsequently, the UDCA residues are observed surrounding the membrane on its hydrophilic side (VDW representation). Notice, how three of them have membrane attraction. Once attached, the UDCA residues, in blue and magenta, remain that way throughout the trajectory.

The third residue of UDCA (metallic blue VDW), moves between the water molecules, but once it is attracted by the membrane, it remains attached to that region.

The last UDCA residue (VDW cyan) is not attracted by the membrane at any time and stays away during the entire trajectory.

*Notes*. The water box and the representations of the UDCA residues are changed from VDW to CPK. The membrane was built with the CHARMM-GUI.ORG server. The structure of UDCA was obtained from the protein data bank (rcsb.org). The video was made with the VMD visualization program (https://www.ks.uiuc.edu/Research/vmd/). The trajectory is approximately 120 ns.
